# Supplementary material for: Intrinsic Expression of Coagulation Factors and Protease Activated Receptor 1 (PAR1) in Photoreceptors and Inner Retinal Layers
Source: Int J Mol Sci. 2022 Jan 17;23(2):984. doi: 10.3390/ijms23020984 (PMC8778890; doi:10.3390/ijms23020984)
Supplement: Supplementary file 1 [file ijms-23-00984-s001.zip › Supplementary_par1_manuscript.pdf]

Intrinsic Expression of Coagulation Factors and Protease Activated Receptor 1 (PAR1) in Photoreceptors and Inner Retinal Layers

Supplementary

Table S1: antibodies details

Antigen	Origin	Dilution	Manufacturer	Catalog #
PAR1	mouse	1:50	Novus biologicals	NBP1-71770
Rhodopsin	rabbit	1:50	Abcam	ab74724
Opsin, red/green	rabbit	1:100	Millipore	ab5405
Opsin, blue	rabbit	1:100	Millipore	ab5407
Secondary antibodies	Origin	Dilution	Manufacturer	Catalog #
Anti-mouse CY3 conjugated	donkey	1:100	Millipore	AP192C
Anti-rabbit IgG H&L (TITC)	Goat	1:100	Abcam	ab6717

Table S2: Tested antibodies directed against PAR1

Origin	Manufacturer	Catalog #
rabbit	Bioss	Bs-0828R
rabbit	Abcam	ab32611
rabbit	MyBioSource	MBS273633

Table S3: Primers

Gene	Forward	Reverse
FPRT	GATTAGCGATGATGAACCAGGTT	CCTCCCATCTCCTTCATGACA
PAR1	GCCTCCATCATGCTCATGAC	AAAGCAGACGATGAAGATGCA
FX	GTGGCCGGGAATGCAA	AACCCTTCATTGTCTTCGTTAATGA
PT (prothrombin)	CCGAAAGGGCAACCTAGAGC	GGCCCAGAACACGTCTGTG

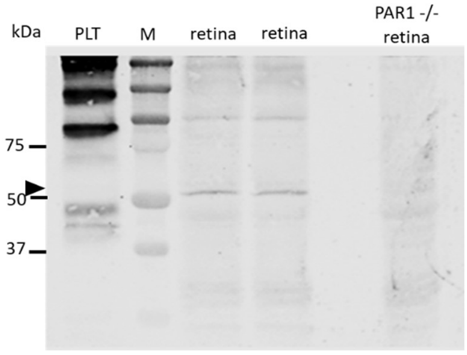

Supplementary figure S1. Uncropped Western blot gel (WB) analysis of PAR1 confirms antibody specificity. *WB was performed with same antibody used for immunofluorescence analysis (NBP-71770, Nuvos biologicals). A ~52kDa protein was detected in lysates of retinas from C57BL/6J mice and platelets (PLT) but not in retina from PAR1 knockout mice (PAR1 $^{-/-}$) or mice platelets. Two representative retinal lysates are shown ("retina"). An empty lane was used to ensure no leakage between lanes.*
